# Early OXA-48-Producing *Enterobacterales* Isolates Recovered in a Spanish Hospital Reveal a Complex Introduction Dominated by Sequence Type 11 (ST11) and ST405 Klebsiella pneumoniae Clones

**DOI:** 10.1128/mSphere.00080-20

**Published:** 2020-04-08

**Authors:** Desirèe Gijón, Ana P. Tedim, Aránzazu Valverde, Irene Rodríguez, María-Isabel Morosini, Teresa M. Coque, Marina Manrique, Eduardo Pareja, Raquel Tobes, Patricia Ruiz-Garbajosa, Rafael Cantón

**Affiliations:** a Servicio de Microbiología, Hospital Universitario Ramón y Cajal and Instituto Ramón y Cajal de Investigación Sanitaria (IRYCIS), Madrid, Spain; b Spanish Network for Research in Infectious Diseases (REIPI), Instituto de Salud Carlos III, Madrid, Spain; c Centro de Investigación Biomédica en Red de Epidemiología y Salud Pública (CIBER-ESP), Barcelona, Spain; d Centro de Vigilancia Sanitaria Veterinaria (VISAVET), Universidad Complutense, Madrid, Spain; e Oh no sequences! Research Group, Era7 Bioinformatics, Granada, Spain; Antimicrobial Development Specialists, LLC

**Keywords:** OXA-48, carbapenemase, VIM-1, CTX-M-15, *Enterobacterales*, ST11

## Abstract

Carbapenemase-producing *Enterobacterales* (CPE) have become an important public health concern. In our hospital, VIM enzymes were first detected in 2005, Klebsiella pneumoniae carbapenemase (KPC) enzymes in 2009, and OXA-48 enzymes in 2012. We assess the population biology of the first OXA-48-producing *Enterobacterales* isolates recovered in our hospital (2012 to 2013) where infections by other carbapenemases had been endemic for several years. Over a 21-month period, 71 isolates (61 Klebsiella pneumoniae, 5 Escherichia coli, 2 Klebsiella aerogenes, and 1 each of Enterobacter cloacae, Klebsiella oxytoca, and Citrobacter amalonaticus) recovered from clinical and surveillance specimens from 57 patients (22.8% nonhospitalized) were investigated for OXA-48-like-producing enzymes. Analyses for characterization and determination of the location of the *bla*_OXA-48_ gene, plasmid transferability, sequence, and clonal relatedness were performed. Most of the isolates also coproduced CTX-M-15 (57/71, 80.3%) and/or VIM-1 (7/71, 9.8%). K. pneumoniae was predominantly identified as sequence type 11 (ST11) (63.4%) and ST405 (9.8%) and E. coli as ST540, ST1406, ST3163, and ST4301. The *bla*_OXA-48_ gene was part of Tn*1999.2* located at the *tir* gene of plasmids (ca. ≥50 kb) of the IncL/M group, also carrying *bla*_VIM-1_ and *bla*_CTX-M-15_ genes. We selected one ST11 K. pneumoniae isolate for whole-genome sequencing in which we studied the plasmid containing the *bla*_OXA-48_ gene. This plasmid was compared with indexed plasmids existing in NCBI database by the use of BRIG and MAUVE. Our data suggest a rapid spread of *bla*_OXA-48_ genes between commonly isolated high-risk clones of *Enterobacterales* species, frequently associated with antibiotic resistance. Moreover, the emergence of the multiresistant ST11 K. pneumoniae clone among nonhospitalized patients emphasizes the difficulty of preventing its dissemination into the community.

**IMPORTANCE** We present results of microbiological analysis of the first *Enterobacterales* isolates that were isolated in 2012 in our institution expressing OXA-48 carbapenemase. This enzyme confers resistance to carbapenems, an important group of antibiotics widely used in the hospitals. OXA-48 carbapenemase is currently present in many parts of the world, but it is found particularly frequently in the Mediterranean area. It was disseminated at the Ramón y Cajal Hospital and found to be associated with a particular Klebsiella pneumoniae strain, so-called high-risk clone ST11, which was previously found in our institution in association with other enzymes such as CTX-M-15, VIM-1, and KPC-3. This clone might have acquired a plasmid harboring the *bla*_OXA-48_ gene. Our results point out the importance of local epidemiology in the dissemination and maintenance of multidrug-resistant bacteria.

## Abstract (precis)

We present results of microbiological analysis of the first *Enterobacterales* isolates that were isolated in 2012 in our institution expressing OXA-48 carbapenemase. This enzyme confers resistance to carbapenems, an important group of antibiotics widely used in the hospitals. OXA-48 carbapenemase is currently present in many parts of the world, but it is found particularly frequently in the Mediterranean area. It was disseminated at the Ramón y Cajal Hospital and found to be associated with a particular Klebsiella pneumoniae strain, so-called high-risk clone ST11, which was previously found in our institution in association with other enzymes such as CTX-M-15, VIM-1, and KPC-3. This clone might have acquired a plasmid harboring the *bla*_OXA-48_ gene. Our results point out the importance of local epidemiology in the dissemination and maintenance of multidrug-resistant bacteria.

## INTRODUCTION

Carbapenemase-producing *Enterobacterales* (CPE) isolates have become an important public health concern worldwide. They have been increasingly reported in Europe, particularly in recent years, with current classification of Spain as a country with “interregional spread” of CPE according to the European Centre for Disease Prevention and Control (ECDC) (1–6).

Carbapenemases include enzymes belonging to Ambler classes A (Klebsiella pneumoniae carbapenemase [KPC] type), B (VIM, IMP, and NDM types, among others), and D (mainly OXA-48). The OXA-48 carbapenemase was first identified in 2003 from a Klebsiella pneumoniae clinical isolate recovered in Turkey (7). Since then, OXA-48 producers have increasingly been detected worldwide but particularly in Europe (8, 9). This enzyme weakly hydrolyses carbapenems and spares extended-spectrum cephalosporins (10). OXA-48-producing isolates often carry genes encoding extended-spectrum β-lactamases (ESBLs) or other carbapenemases, hindering their accurate detection, particularly in hospitals with a high prevalence of different carbapenemase enzymes (11).

In our hospital in Madrid (Spain), VIM enzymes were first detected in 2005 (1), KPC enzymes in 2009 (12, 13), and OXA-48 enzymes in 2012. It is of note that large OXA-48 outbreaks were described in other hospitals in Madrid (2) and in other Spanish regions (3, 14, 15) before the emergence in our center. Epidemiology of CPE has been investigated not only locally but also at a national level in the Spanish reference center (16, 17). The aim of this work was to characterize the microbiological and epidemiological scenario, through classical and genomic approaches, represented by the first OXA-48-producing *Enterobacterales* recovered in our hospital (2012 to 2013), where infections by strains producing other carbapenemases (mainly VIM and KPC) had been endemic for several years.

## RESULTS

### Patient characteristics and carbapenemase characterization.

A total of 57 patients (33 males) were infected (*n* = 41) or colonized (*n* = 16) with OXA-48-producing *Enterobacterales* during the studied period. The majority of patients were elderly (median age, 69 years; range, 18 to 92 years). In this period, the prevalence of carbapenemase in our institution was 0.4% and the predominant carbapenemase was OXA-48. Patients were admitted to medical (*n* = 20; 4%) and surgical (*n* = 21; 4%) wards and intensive care units (ICU) (*n* = 4; 7%). It should be noted that 13 isolates were recovered from 13 nonhospitalized patients at the time of sampling, although 5 of them had been previously admitted to our institution (Table 1). The first OXA-48-producing *Enterobacterales* isolates recovered in our institution were obtained from patients admitted to the urology ward in 2012. In that ward, most of the K. pneumoniae isolates belonged to the same clone (KP-A) identified as sequence type 11 (ST11). Overall, from the 57 patients, we recovered 71 isolates (61 K. pneumoniae, 5 Escherichia coli, 2 Klebsiella aerogenes, and 1 each of Klebsiella oxytoca, Enterobacter cloacae, and Citrobacter amalonaticus). Apart from the OXA-48 enzyme, 51 isolates (48 K. pneumoniae, 1 K. oxytoca, 1 K. aerogenes, and 1 C. amalonaticus) coproduced CTX-M-15, 6 isolates (4 K. pneumoniae and 2 E. coli) coproduced VIM-1 and CTX-M-15, and 1 E. coli isolate also coproduced VIM-1. It is of note that the double-disk synergy test (using EDTA) was negative in 5 of 7 isolates harboring VIM-1.

**TABLE 1:** Epidemiological data for OXA-48-producing *Enterobacteriaceae* isolates^*a*^

Species (no. of isolates)	PFGE type (no. of isolates)	Sequence type	Plasmid size(s) (kb)	RFLP pattern	Rep type	Coresistance	Ward(s) and/or patient status	Other β-lactamase
*K. pneumoniae* (*n* = 61)	A (*n* = 40)	11	50 (*n* = 38)	A	L/M (*n* = 17)	Gm, Tb, Cp, Nx, S/T, Fm, Nf	Urology (*n* = 10), general surgery (*n* = 5), pneumology (*n* = 1), vascular surgery (*n* = 1), preventive medicine (*n* = 5), internal medicine (*n* = 3), hematology (*n* = 2), cardiology (*n* = 1), gastroenterology (*n* = 1), nephrology (*n* = 1), neurosurgery (*n* = 2), ICU (*n* = 2), outpatients (*n* = 6)	CTX-M-15 (*n* = 38), VIM-1 (*n* = 3)
			220 (*n* = 2)	ND	FIIk (*n* = 2)			
	D (*n* = 2)	11	50 (*n* = 1)	A	L/M (*n* = 1)	Tb (*n* = 2), Cp (*n* = 2), Gm (*n* = 1), Fm (*n* = 1)	Outpatients (*n* = 2)	CTX-M-15 (*n* = 2)
			60 (*n* = 1)	ND				
	I (*n* = 1)	11	65	A	L/M	Gm, Tb, Cp, S/T	Hematology (*n* = 1)	CTX-M-15
	K (*n* = 1)	11	60	A		Gm, Tb, Cp, S/T	General surgery (*n* = 1)	CTX-M-15
	M (*n* = 1)	11	65	A		Tb, Nx, Cp, S/T, Fm	Outpatient (*n* = 1)	CTX-M-15
	F (*n* = 1)	15	40	B	L/M	Cp, Fm	Pneumology (*n* = 1)	
	H (*n* = 1)	15	50	B		Gm, Cp, S/T	Outpatient (*n* = 1)	
	O (*n* = 1)	307	60			Gm, Tb, Cp, S/T	Otorhinolaryngology (*n* = 1)	CTX-M-15 (*n* = 2)
	P (*n* = 1)	307	60				Neurosurgery (*n* = 1)	
	B (*n* = 5)	405	50 (*n* = 2)	C (*n* = 3)	L/M (*n* = 4)	Gm (*n* = 5), Tb (*n* = 5), Cp (*n* = 5), S/T (*n* = 5), Fm (*n* = 1)	Vascular surgery (*n* = 2)	CTX-M-15 (*n* = 4)
			60 (*n* = 2)				General surgery ICU (*n* = 2)	
			70 (*n* = 1)				Outpatient (*n* = 1)	
	E (*n* = 2)	405	60 (*n* = 1)	ND	L/M (*n* = 1)	Gm (*n* = 1), Tb (*n* = 1), Cp (*n* = 1), S/T (*n* = 2)	Geriatrics (*n* = 1)	CTX-M-15 (*n* = 1)
			40 (*n* = 1)				Gastroenterology (*n* = 1)	
	J (*n* = 1)	405	60	C	L/M	Gm, Tb, Cp, S/T	Urology (*n* = 1)	CTX-M-15 + VIM-1
	C (*n* = 1)	487	50		L/M		Traumatology (*n* = 1)	
	G (*n* = 1)	712	60	A	L/M		Internal medicine (*n* = 1)	CTX-M-15
	N (*n* = 1)	971	50	A			Oncology (*n* = 1)	
	L (*n* = 1)	1427				Gm, Tb, Cp, S/T, Fm	Outpatient (*n* = 1)	

*E. coli* (*n* = 5)	C (*n* = 1)	540	50	D	L/M	S/T	Outpatient (*n* = 1)	
	B (*n* = 2)	1406	60	A	L/M (*n* = 2)	Gm (*n* = 1), Tb (*n* = 1), S/T (*n* = 2)	General surgery (*n* = 2)	CTX-M-15 (*n* = 1)
				ND				VIM-1 (*n* = 2)
	A (*n* = 1)	3163	50	ND		Gm, Tb, Cp, S/T	Urology (*n* = 1)	CTX-M-15 + VIM-1
	D (*n* = 1)	4301	50	A	L/M	Cp, S/T	Oncology (*n* = 1)	

*K. aerogenes* (*n* = 2)			50	ND	L/M (*n* = 2)	Gm (*n* = 1), Tb (*n* = 1), Cp (*n* = 1), S/T (*n* = 1)	Urology (*n* = 1), general surgery ICU (*n* = 1)	CTX-M-15 (*n* = 1)

*E. cloacae* (*n* = 1)			50	A	L/M	Gm, Tb, Cp, S/T	Traumatology (*n* = 1)	

*K. oxytoca* (*n* = 1)			50		L/M		Oncology (*n* = 1)	CTX-M-15

*C. amalonaticus* (*n* = 1)			50	A	L/M		Infectious disease (*n* = 1)	CTX-M-15

a Abbreviations: ICU, intensive care unit; Gm, gentamicin; Tb, tobramycin; Cp, ciprofloxacin; NX, nalidixic acid; S/T, trimethoprim-sulfonamide; Fm, fosfomycin; NF, nitrofurantoin.

### Antibiotic susceptibility.

All OXA-48-positive isolates were resistant to β-lactam–β-lactamase inhibitor combinations and to broad-spectrum cephalosporins. However, some OXA-48-positive isolates (43/71, 60.5%) were susceptible to imipenem (41/71, 58%) and ertapenem (2/71, 3%). Phenotypes of resistance to non β-lactam antibiotics are presented in Table 1. Most of the isolates were resistant to gentamicin (52/71, 73%), tobramycin (56/71, 79%), ciprofloxacin (61/71, 86%), and trimethoprim-sulfamethoxazole (60/71, 84.5%).

### Clonal background.

**FIG 1:**
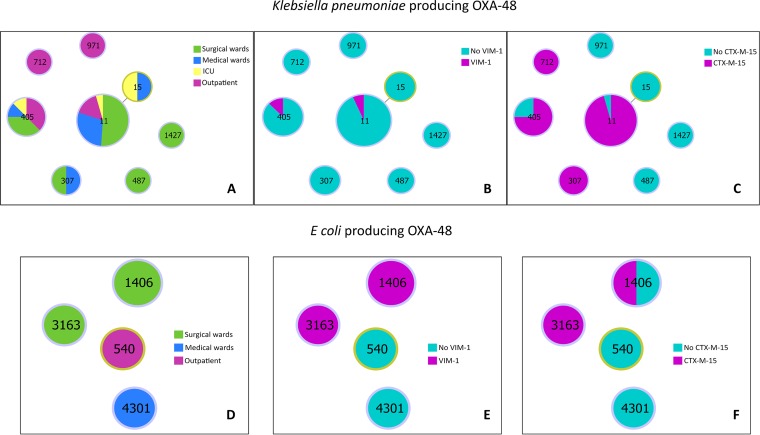
Results of goEburst analysis of K. pneumoniae (A to C) and E. coli (D to F) producing OXA-48 isolates differentiated by isolate collection location (A and D), presence or absence of VIM-1 (B and E), and presence or absence of CTX-M-15 (C and F).

OXA-48-producing K. pneumoniae isolates were classified in 16 pulsed-field gel electrophoresis (PFGE) types (KP-A to KP-P) and 7 sequence types (ST), namely, ST11, ST15, ST307, ST405, ST487, ST712, and ST971 (Fig. 1). ST11 and ST405 were the STs most frequently found, recovered from samples collected from patients in different hospital wards, and K. pneumoniae isolates coproducing VIM-1 and CTX-M-15 belonged to these STs (Table 1). Carbapenemase-producing (CP) E. coli isolates were classified in four distinct PFGE types identified as ST540, ST1406, ST4301, and ST3163, the last being described for the first time in this report. The E. coli isolates coproducing OXA-48 and VIM-1 belonged to ST1406 (*n* = 1), and the isolates coproducing OXA-48, VIM-1, and CTX-M-15 (*n* = 2) belonged to ST1406 and ST3163 (Fig. 1).

### Transferability and location of *bla* carbapenemase genes and plasmid typing.

The *bla*_OXA-48_ gene was located on plasmids (50 to 70 kb), most of them being transferable by conjugation (59/71 isolates). They carried *bla*_OXA-48_, often with *bla*_VIM-1_ (7/71, 9.8%) and/or *bla*_CTX-M-15_ (57/71, 80.3%). OXA-48-carrying plasmids were categorized as IncL/M according to the PCR-based replicon typing (PBRT) scheme (18). In addition, 2 of 71 isolates presented a larger (ca. 220-kb) plasmid nonrelated to *bla* genes and typed as IncFIIk by the scheme described previously by Villa et al. (19) (Table 1). Restriction fragment length polymorphism (RFLP) analysis for OXA-48 plasmids revealed a similar band pattern (Fig. S1). These results indicate a similar backbone of the OXA-48 encoding plasmids, a finding also supported by the results produced by PCR and sequencing of their *repA*, *traU*, and *parA* genes (Table 1). More than 98% identity among all these plasmids and with the pOXA-48 plasmids described previously by Poirel et al. (20) was observed.

10.1128/mSphere.00080-20.1 FIG S1 Restriction fragment length polymorphisms. Download FIG S1, TIF file, 2.1 MB. Copyright © 2020 Gijón et al. 2020 Gijón et al. This content is distributed under the terms of the Creative Commons Attribution 4.0 International license.

### Characterization of pRYC-OXA-48.

**FIG 2:**
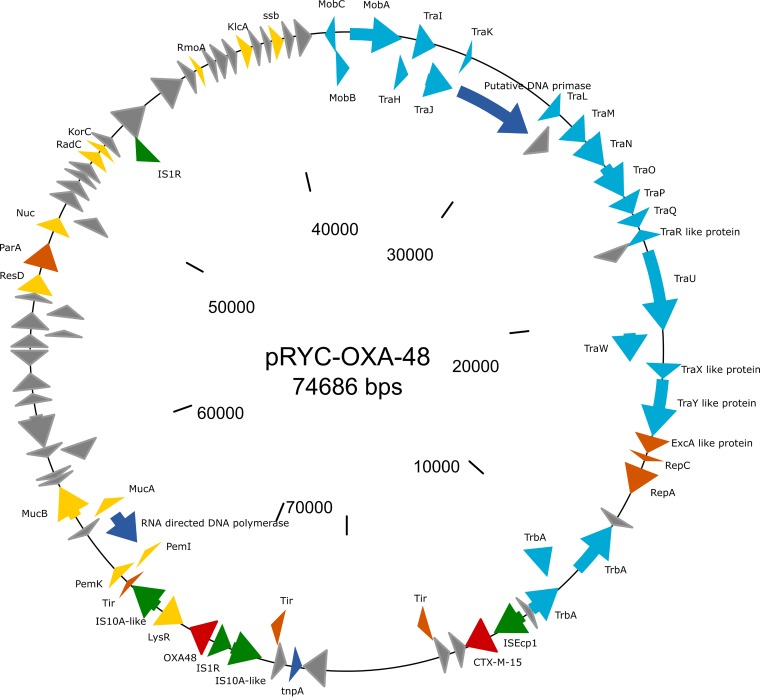
ST11 K. pneumoniae clone carrying pRYC-OXA48.

**FIG 3:**
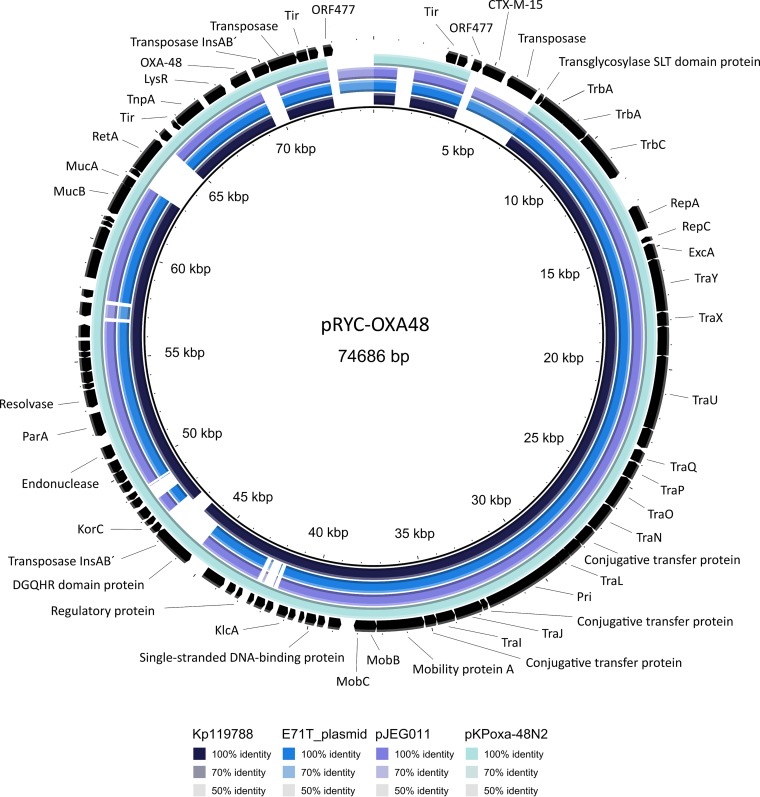
Brig comparison of pRYC-OXA-48 with other OXA-48-carrying plasmids.

The plasmid isolated from strain F64-ST11-OXA-48 was an IncL/M plasmid of 74,686 bp, comprising 92 coding DNA sequences (CDS; GC%, 50.8%) and containing two *bla* genes (*bla*_OXA-48_ and *bla*_CTX-M-15_) as the only antibiotic resistance markers. The *bla*_OXA-48_ gene was part of Tn*1999.2* inserted at the *tir* gene, responsible for inhibition of transfer of the plasmid, whereas the *bla*_CTX-M-15_ gene was located downstream of IS*Ecp1* (Fig. 2). The resistance region of the plasmid (including both *bla* resistance determinants) was delimited by two insertion sequences: IS*Ecp1* was found to be associated with the *bla*_CTX-M-15_ gene and an IS*10A*-like sequence located upstream of *bla*_OXA-48_ (Fig. 2). *In silico* comparisons with other OXA-48-encoding-plasmids revealed a very similar structure (>99% similarity) with the same backbone as that found in the IncL-OXA-48 plasmids (Fig. 3) (GenBank accession no. JN626286, KC335143, KC354801, and NC_021502).

## DISCUSSION

Spread of CPE has been increasingly reported worldwide since first description more than 20 years ago, with predominance of metallo-beta-lactamase (MBL) producers in Asia and Europe, KPC producers in the United States, and OXA-48 producers in the Mediterranean countries (6, 9, 21, 22). These carbapenemases have been increasingly recovered in Spain, and OXA-48 isolates are currently the most prevalent ones (3, 6). In our study, the prevalence of carbapenemase in our institution was 0.4%, and the most prevalent carbapenemase was OXA-48 (50.3%). The carbapenemase situation in our institution, which started with VIM producers in 2005 and KPC producers in 2009 (1, 12, 13), can be observed in Fig. S2. In this article, we characterize the early isolates of OXA-48-producing *Enterobacterales* collected in our hospital; currently, the situation in our institution is one of OXA-48 infection endemicity (11). Due to the difficulties in the detection of OXA-48 carbapenemase producers by the use of carbapenem susceptibility results as a screening test, we implemented measurement of increases in temocillin MIC as a surrogate marker to detect its presence in CarbaNP test-positive isolates with a negative result for MBL or KPC enzymes (23, 24). Nevertheless, we recovered isolates with decreased susceptibility to carbapenemase to perform specific PCR for OXA-48, and results were negative (data not shown).

10.1128/mSphere.00080-20.2 FIG S2 Epidemiological curve of carbapenemasas in Hospital Universitario Ramón y Cajal (2005 to 2013). Download FIG S2, TIF file, 1.1 MB. Copyright © 2020 Gijón et al. 2020 Gijón et al. This content is distributed under the terms of the Creative Commons Attribution 4.0 International license.

Isolation of OXA-48-producing *Enterobacterales* in our hospital started in the urology ward in 2012 (Fig. S2). In that ward, most of the K. pneumoniae isolates belonged to the same clone (KP-A) which corresponded to ST11. Interestingly, this ST was previously found in our institution in 2010 in a patient admitted to general surgery who was infected with a KPC-3-producing K. pneumoniae strain and in 2011 in 2 patients admitted to the neurosurgery ICU who were colonized with a VIM-1-producing K. pneumoniae strain. These isolates belonged to the same ST and showed closely related PFGE patterns, suggesting that the circulation of a clonal local pool in our institution plays an important role in the emergence and spread of new resistant variants, as happened with the OXA-48-producing clone. Later, the same clone was detected in other wards in addition to ST405 K. pneumoniae. The latter was first identified at the nearby Hospital La Paz (500 m from our hospital) where such infections had become endemic (2). However, in spite of the close proximity of the two hospitals, these ST405 isolates were found to have different PFGE patterns (data not shown), suggesting independent introduction events. In a study published in 2017 by the national reference laboratory that included data representing K. pneumoniae isolates collected in different Spanish areas, both sequence types were found among the most prevalent ones, highlighting their persistence over time in our country (25).

We detected the presence of *bla*_CTX-M-15_ in most OXA-48-producing isolates. It is of note that we previously found CTX-M-15 in ST11 K. pneumoniae isolates recovered from rectal swabs (data not published), denoting possible acquisition of *bla*_OXA-48_ determinants in circulating clones in our institution. Coproduction of OXA-48 and CTX-M-15 in *Enterobacterales* has been widely described previously (26, 27). However, in pRYC-OXA-48, *bla*_CTX-M-15_ was associated with IS*Ecp1*, but without interrupting the Tn*1999.2* which contains *bla*_OXA-48_ (Fig. 2), a configuration also previously described (28–30).

It should be noted that 7 isolates (4 K. pneumoniae and 3 E. coli) coproduced VIM-1 and that 6 of them (4 K. pneumoniae and 2 E. coli) also coproduced CTX-M-15. The presence of more than one *bla* gene in these isolates can be explained by the carbapenemase endemic situation involving *bla*_VIM_, *bla*_KPC_ and *bla*_CTX-M-15_ genes in our hospital (1, 11, 13). The presence of both *bla*_VIM-1_ and *bla*_OXA-48_ enzymes in the same isolate enhances the difficulty of phenotypically detection of OXA-48 producers. We believe that in these cases, the double-disk synergy test performed with EDTA is not entirely reliable to identify VIM-1 producers as in our study we had five out of seven false-negative results.

To our knowledge, there have been only limited previously published descriptions of the coexistence of OXA-48 and VIM-1 (31–34), but the availability of such reports might increase in the future. Interestingly, we observed that *bla*_OXA-48_ and *bla*_VIM-1_ genes, mostly associated with K. pneumoniae ST11, were located on the same plasmid. The plasmids detected in our study (among different PFGE patterns) showed a high degree of similarity (>99%) with previously described OXA-48 plasmids, even with those also encoding *bla*_VIM-1_ and *bla*_CTX-M-15_. This indicates that the spread of genes encoding this carbapenemase, which are often linked with other beta-lactamase genes, is associated not only with the appearance of highly adapted clones in the hospital but also with the dissemination of highly transmissible plasmids. The presence of these plasmids in the hospital setting allows acquisition and rapid spread of new resistance determinants such as *bla*_OXA-48_. The characterized IncL/M plasmid is currently present in our institution (31).

In conclusion, our data showed rapid penetration and spread of *bla*_OXA-48_ genes in multiresistant clones of *Enterobacterales* after its emergence in our institution. The globally spread ST11 K. pneumoniae clone, previously associated in our hospital with CTX-M-15, VIM-1, and KPC-3 enzymes, is now also present coproducing OXA-48 (11). This scenario reinforces the idea of the contribution of high-risk clones in the dissemination and persistence of antibiotic resistance genes. Moreover, emergence of multiresistant ST11 among nonhospitalized patients highlights the difficulties in preventing dissemination of such strains into the community.

## MATERIALS AND METHODS

### Bacterial isolates.

Over a 21-month study period (March 2012 to December 2013), all isolates (*n* = 71) recovered from clinical specimens (27 urine samples, 9 wounds, 8 sterile fluids, 5 respiratory samples, 2 blood cultures) and surveillance cultures (20 rectal swabs) suspected of carbapenemase production but negative for the phenotypic expression of classes A and B carbapenemases (see below) were investigated for OXA-48-like-producing enzymes. Patients´ demographic characteristics were also reviewed. The study was approved by the ethical committee of our institution (reference 251-13). For clonal comparison, we also included in the analysis the CPE clinical isolates associated with contemporary outbreaks due to strains producing MBL or KPC in our institution (1, 12, 13) and to OXA-48 producers collected in a nearby hospital (Hospital La Paz, 500 m from our hospital) (2).

### Bacterial identification, susceptibility testing, and phenotypic assays.

Species identification and antibiotic susceptibility testing were performed using matrix-assisted laser desorption ionization–time of flight (MALDI-TOF) (Bruker Daltonics, Leipzig, Germany) and the MicroScan automated system (Beckman Coulter, Brea, CA), respectively. MICs were interpreted using EUCAST criteria (www.eucast.org). Screening for the presence of carbapenemase production was performed with CarbaNP (23). MBL production was investigated by a double-disk synergy test using EDTA plus meropenem and ceftazidime disks as previously described (1, 35). KPC production was inferred by an increase of the inhibition zone of an unsupplemented meropenem disk compared with that of a meropenem disk supplemented with 0.3 mg of boronic acid (36). In addition, OXA-48 production was phenotypically inferred by the lack of the inhibition zone of the temocillin disk (37).

### Characterization of *bla* genes and clonal relatedness.

The presence of genes encoding carbapenemase (*bla*_OXA-48_, *bla*_VIM_, and *bla*_KPC_) and ESBLs (*bla*_TEM_, *bla*_SHV_, and *bla*_CTX-M_) classes was screened by PCR as previously described (1, 38). Clonal relatedness was established by pulsed-field gel electrophoresis (PFGE) analysis of XbaI (New England Biolabs, Inc., England)-digested genomic DNA (39). Multilocus sequencing typing (MLST) was performed for K. pneumoniae isolates (https://bigsdb.pasteur.fr/klebsiella/klebsiella.html) and E. coli isolates (http://mlst.warwick.ac.uk/mlst/dbs/Ecoli).

### Transferability and location of *bla* carbapenemase genes and plasmid typing.

Mating experiments were performed using E. coli strain BM21 (nalidixic acid and rifampin resistant, lactose fermentation positive, and plasmid free) as the recipient (1). The plasmid content (number and size) of each strain was analyzed by S1-digested genomic DNA PFGE, the *bla* gene location being detected by standard hybridization procedures (40, 41). Transconjugants were selected on Luria-Bertani agar plates containing imipenem (0.5 μg/ml) and rifampin (100 μg/ml) and were incubated at 37°C for 24 h. Plasmid incompatibility groups were inferred by PCR typing schemes based on the presence of genes encoding replication initiator proteins (RIP) (18, 19). Further characterization of OXA-48 gene-carrying plasmids was performed for all K. pneumoniae PFGE types by comparison of restriction fragment length polymorphism (RFLP) patterns of DraI-digested plasmid DNA. To further characterize the OXA-48 IncL/M plasmids, the genes associated with replication (*repA*), conjugation (*traU*), and maintenance (*parA*) were amplified and further sequenced (20).

### ST11 whole-genome sequencing and plasmid characterization.

K. pneumoniae ST11 strains associated with production of either VIM-1 (31) or KPC (13) have been prevalent in our hospital since their first detection in 2005. Due to the high transmissibility and persistence of this clone (11, 42), we sequenced one ST11 K. pneumoniae isolate, which was selected based on its ESBL-positive phenotype (this isolate coproduced both OXA-48 and CTX-M-15). Whole-genome sequencing (WGS) was performed using Illumina (280× coverage), and DNA fragments were subjected to *de novo* assembly with Velvet and Spades (43). In addition, the first ST11–OXA-48 isolate found in our hospital (ryc_12106801) was also sequenced and assembled using PacBio and RS_HGAP Assembly 2, respectively. Combining the sequences obtained by Illumina and PacBio, and using SPAdes, the plasmid of this isolate was closed (44–47). The *in silico* plasmid typing was carried out using the PlasmidFinder tool (48). This plasmid was compared with other indexed plasmids at the NCBI database (GenBank accession numbers JN626286.1, KC335143.1, KC354801.1, and NC_021502.1) using BRIG (49) (http://brig.sourceforge.net/) and MAUVE (http://darlinglab.org/mauve/mauve.html).

### Data availability.

Data representing this Whole Genomes Shotgun project have been deposited at DDBJ/ENA/GenBank under accession number VILG00000000. The version described in this paper is version VILG01000000.
