# Supplementary figures and images for: Early OXA-48-Producing Enterobacterales Isolates Recovered in a Spanish Hospital Reveal a Complex Introduction Dominated by Sequence Type 11 (ST11) and ST405 Klebsiella pneumoniae Clones
Source: mSphere. 2020 Apr 8;5(2):e00080-20. doi: 10.1128/mSphere.00080-20 (PMC7142293; doi:10.1128/mSphere.00080-20)

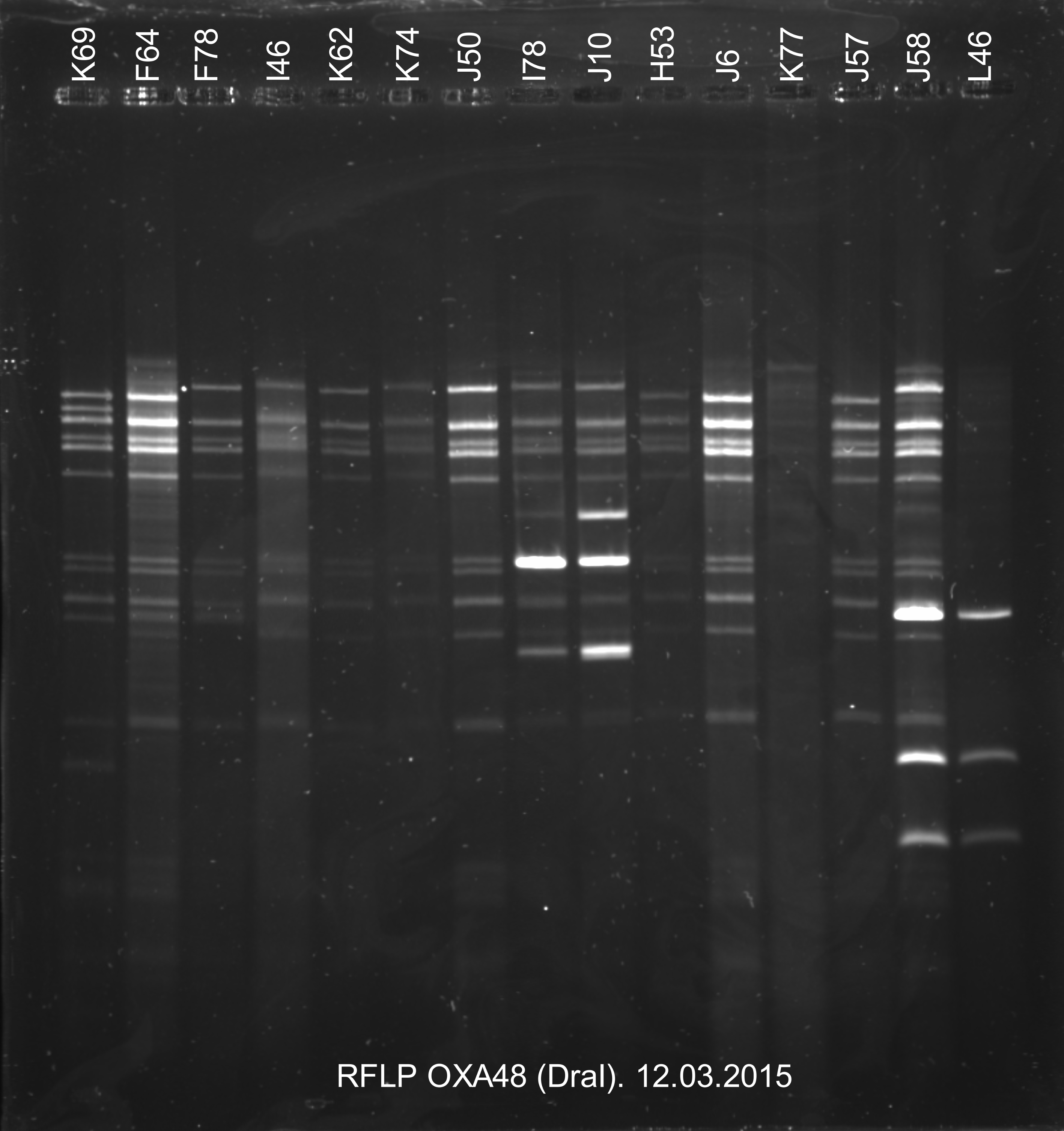

Supplement: FIG S1 [file mSphere.00080-20-sf001.tif]

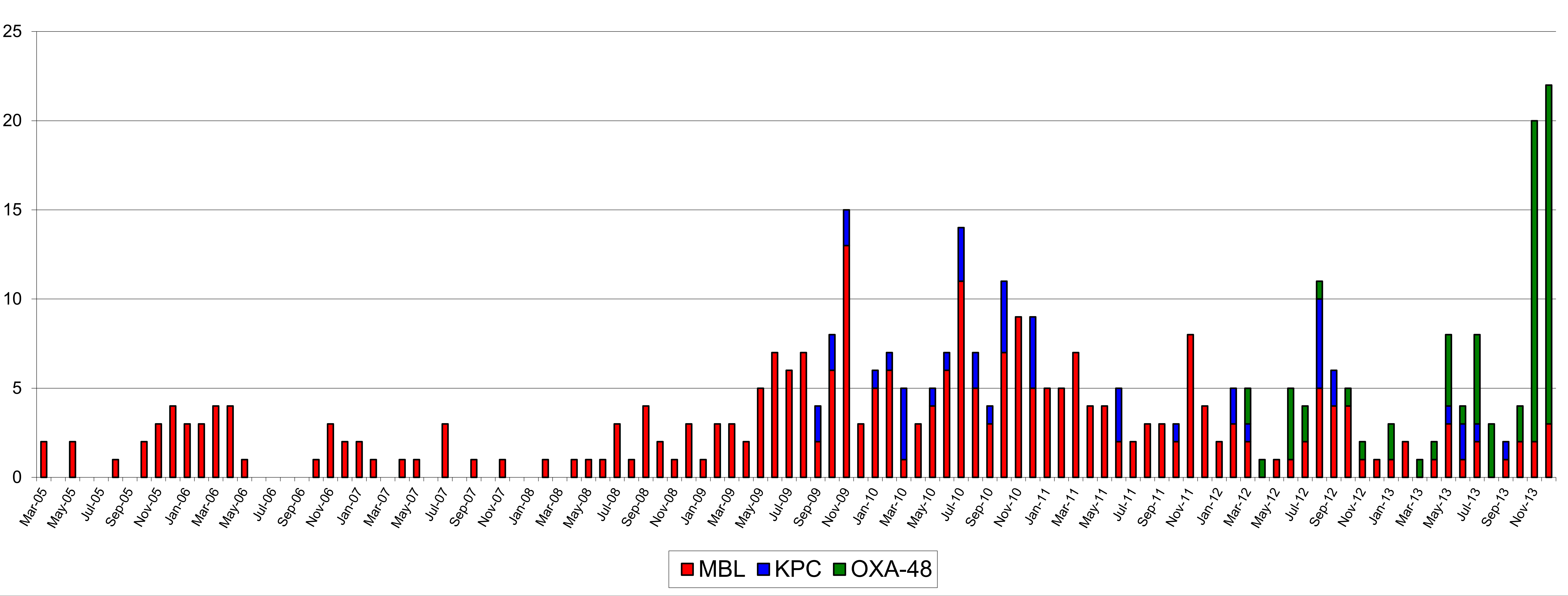

Supplement: FIG S2 [file mSphere.00080-20-sf002.tif]
